# Impact of lymphocyte infiltration on the survival of patients with gastric and colorectal cancers at the Yaoundé General Hospital (Cameroon)

**DOI:** 10.11604/pamj.2025.50.79.43589

**Published:** 2025-03-18

**Authors:** Etienne Okobalemba Atenguena, Astryde Larissa Tchutchou Ndjeutcham, Vanelle Lotie Messah Kamdem, Estelle Alida Ngne Mbopda, Manuella Mayemi, Carole Marlise Menzy, Stéphane Zingue

**Affiliations:** 1 Oncology Division, Yaoundé General Hospital, Yaoundé, Cameroon,; 2 Department of Internal Medicine, Faculty of Medicine and Biomedical Sciences, University of Yaoundé I, P.O. Box 1364 Yaoundé, Cameroon,; 3 School of Health Sciences, Catholic University of Central Africa, Yaoundé, Cameroon,; 4 Department of Pharmacotoxicology and Pharmacokinetics, Faculty of Medicine and Biomedical Sciences, P.O. Box 1364, Yaoundé, Cameroon

**Keywords:** Lymphocyte infiltration, survival, digestive cancer, colorectal cancer, gastric cancer

## Abstract

**Introduction:**

digestive cancers (DC) are a group of cancers affecting the gastrointestinal tract and are capable of triggering an immune response. The cells produced during this response are tumor effectors whose role is to rid the body of tumor cells. The functional role of these cells, particularly the tumor-infiltrating lymphocytes (TILs), in the prognosis of patients remains poorly understood in Cameroon. This study aimed to evaluate the impact of lymphocyte infiltration on the survival of patients with certain digestive cancers.

**Methods:**

we conducted a retrospective cross-sectional study at the Oncology Department and the Anatomo-Cytopathology Laboratory of the Yaoundé General Hospital (YGH). Patients histologically diagnosed with colorectal and gastric cancers with available data from YGH between 2019 and 2023, who consented to participate and had a biopsy sample available at the YGH laboratory, were included in the study. Initially, we described patients' sociodemographic, clinical, and pathological characteristics. Then, we estimated the grade of lymphocyte infiltration in colorectal and gastric cancers using Hematoxylin-Eosin (HE) staining and analyzed the correlation between lymphocyte infiltration and patient survival through Cox regression. Data were analyzed with a significance level set at 5% for all comparisons.

**Results:**

the study enrolled 90 patients with colorectal cancer and 50 with gastric cancer. Overall survival was 64.8% at 49 months in the study population, with the median not reached for colorectal cancer, and 64% at 39 months for gastric cancer, also with a median not reached. The average age at diagnosis for colorectal and gastric cancers was 54 ± 14.53 years and 53.24 ± 11.41 years, respectively. Men predominated in both pathologies, with a sex ratio of approximately 1.11. Colonic location was predominant (53%; 46/90) for colorectal cancer, with stage III disease being most common, while the antropyloric location (46%; 23/50) was predominant for gastric cancer, with stage IV being most frequent according to the AJCC. Moreover, 86% of patients had TILs in their histological samples, with a predominance of high TILs in both colorectal (38%; 34/90) and gastric (42%; 21/50) cancers. The performance of chemotherapy was inversely proportional to TILs in colorectal cancer, while no significant difference was found between TILs and chemotherapy in gastric cancer. However, no association was found between TILs and patient survival in either colorectal or gastric cancers. Patients who had metastases had a risk of death of 14.07 (aHR: 14.07, 95% CI 1.66-119.24; p = 0.015) compared with those who did not. Similarly, patients who had not taken chemotherapy had a 21.32 greater risk of death (aHR: 21.32, 95% CI 5.35-84.96; p<0.001) than those who had.

**Conclusion:**

there was no statistically significant difference in survival between patients suffering from colorectal and gastric cancers, and the grade of lymphocyte infiltration. Survival was significantly impacted by the presence of metastasis and the absence of treatment.

## Introduction

Digestive cancer (DC) refers to a group of cancers affecting organs and structures within the digestive system, including the esophagus, stomach, liver, pancreas, small intestine, colon, and rectum. These cancers can develop when cells in any part of the digestive system grow and divide abnormally [1]. Among them, colorectal cancer and gastric cancer are the most common primary cancers of the digestive tract. Colorectal cancer ranks third among the most frequently diagnosed cancers worldwide, while gastric cancer ranks fifth [2].

Tumor-infiltrating lymphocytes (TILs) have been a subject of fascination in oncology for many years, particularly in the context of colorectal and gastric cancers. These infiltrating lymphocytes have been shown to significantly impact the survival outcomes of patients [3]. Studies by Galon *et al*. [4] on digestive cancers demonstrated a major prognostic impact due to lymphocyte infiltration, independent of TNM classification. Understanding the role of lymphocyte infiltration in digestive cancer is crucial for improving patient prognosis and developing targeted therapeutic approaches. Although the presence of lymphocyte infiltration in DC has been described in numerous studies, few of these studies have extended to our Cameroonian context.

In light of the above, it is imperative to evaluate the impact of lymphocyte infiltration on the survival of patients with DC at the YGH to enhance treatment and management strategies, and to develop appropriate, effective, and sustainable solutions to improve 5-year survival rates in Cameroon. It is within this context that the present study falls, which will shed light on a new prognostic tool that could overcome the limitations of the current TNM classification of colorectal cancer in our Cameroonian setting. Thus, this study aimed to evaluate the impact of lymphocyte infiltration on the survival of patients with gastric and colorectal cancers.

## Methods

**Study design:** this study is a retrospective cross-sectional study conducted over 11 months from January to November 2023. The proportion of patients histologically diagnosed with digestive cancer with available data was determined. Patients were described based on their sociodemographic, clinical, and pathological characteristics, as well as the histology of their biopsy specimens. Lymphocyte infiltration rates were graded. Multivariate analyses were performed using Cox regression to determine the statistical significance of associations between the systemic treatment received (chemotherapy) and the grade of tumor-infiltrating lymphocytes (TILs). Lymphocyte infiltration grades were analyzed as categorical variables. Patient survival was estimated using the Kaplan-Meier method, and survival curves were compared using the log-rank test. The 5-year survival was calculated as the time elapsed between the date of cancer diagnosis and the date of death, loss of follow-up, or last follow-up.

**Setting:** the Yaoundé General Hospital (YGH) is one of the most specialized hospitals for cancer treatment in Cameroon. It offers various specialized services including Radiotherapy, Medical Oncology, Anatomic Pathology, Nuclear Medicine, Gynecology, and Surgery. Patients with digestive cancers (DC) receiving care at YGH are often referred from other health facilities across the country.

**Participants:** digital medical records of patients with digestive cancer (colorectal and gastric) followed at the YGH between 2019 and 2023 were used. All medical records of patients included in this study during the 5-year study period were reviewed.

**Variables:** data collected and analyzed included: sociodemographic characteristics (age, education level, religion, marital status, ethnolinguistic area, and menopause); clinical characteristics (affected breast, histological type, histological grade, type of treatment, AJCC stage, consistency of discovery); different grades of TILs.

**Data sources/measurement:** sampling was conducted using a non-probabilistic convenience method, enrolling only patients meeting the criteria outlined in our study. Between 2019 and 2023, we collected data from 90 patients diagnosed with colorectal cancer and 50 patients diagnosed with gastric cancer histologically and treated at YGH. Patients with inaccessible files or unavailable biopsy documents, as well as those who declined participation, were excluded.

**Bias:** all medical records of colorectal and gastric cancer patients arriving at the hospital during the study period were included to avoid bias.

**Quantitative variables:** sections and biopsies of colorectal and gastric pieces were analyzed after staining with hematoxylin-eosin (H&E 200x and 400x) under a light microscope by a pathologist. The TILs evaluated were those located in the tumor stroma zone. Using a light microscope, the rate of lymphocytic infiltration in tumor and peritumoral tissue was determined as a percentage in the observed area and defined as follows: absent (0%), low TIL (≤10%); intermediate TIL (10-59%) and high TIL (≥60%). Only stromal TILs have been considered in this study; TILs present in areas with grinding artifacts, necrosis, and inflammation around biopsy sites were not taken into account.

**Data analysis:** once our data matrix had been obtained, it was transferred to Epi Info 7 software for statistical analysis. We report descriptive categorical data with percentages and descriptive numerical data with mean and standard deviation. A p-value below 0.05 was considered statistically significant. Survival was calculated by the Kaplan-Meier method using SPSS software, and hazard ratios were calculated by Cox proportional Hazard regression with a 95% confidence interval.

**Ethical consideration:** study protocols were approved by the institutional ethics committee of the Catholic University of Central Africa, Cameroon. Patient consent was required for this cross-sectional study.

## Results

**Participants:** in this study 145 patients with colorectal cancer were recruited, 55 of whom were excluded (8 who failed to give informed consent, 32 whose paraffin blocks were unusable, and 15 with incomplete medical records) affording a final sample size of 90. In the same period, 90 gastric cancer patients were diagnosed and managed at the YGH, and 08 refused to give informed consent. From the 82 files recorded, 11 patients had incomplete medical records and 21 unavailable biopsies, giving us a final sample size of 50.

**General characteristics of the study population:** the results depicted in Table 1 show that the mean age of gastric cancer patients was 53.24 ± 11.41 years, with a minimum age of 21 years and a maximum of 74 years. The most represented age group was 50 to 64 years old, comprising 54% of the cases. The majority of the sex was male, accounting for 28 cases (56%). The tumor was most commonly encountered at the antropyloric level (46%; 23/50) followed by the fundal level (44%; 22/50). Stage IV was the most represented stage according to the AJCC classification, accounting for 54% of cases (27/50). Table 2 summarizes the socio-demographic characteristics of patients with colorectal cancer. The age of the population ranged from 21 to 84 years, with a mean age at diagnosis of 54 ± 14.53 years. The most represented age group was 50 to 64 years old, and the majority sex was male, with 47 cases (53%). Most tumors were located in the colon (53%; 46/90). Stage III was the most represented stage according to the AJCC classification, accounting for 42% of cases (38/90), followed by stage IV with a proportion of 33% (30/90).

**Table 1:** summary of sociodemographic characteristics of patients with gastric cancer

Variables	Frequency	Percentage (%)
**Age**		
20-34	5	10.0
35-49	8	16.0
50-54	27	54.0
65-74	10	20.0
**Sex**		
Male	28	56.0
Female	22	44.0
**Location**		
Antropyloric	23	46.0
Fundus	22	44.0
Great curvature	3	6.0
Small curvature	2	4.0
**AJCC stage**		
I	11	22.0
II	09	18.0
III	3	6.0
IV	27	54.0
**Treatment**		
Yes	45	90.0
No	5	10.0
**Surgery**		
Yes	10	20.0
No	40	80.0
**Lymphocyte infiltration**		
Yes	45	90.0
No	5	10.0
**Grade of TILs**		
Absent	5	10.0
Weak	8	16.0
Moderate	16	32.0
High	21	42.0
**Metastasis**		
Yes	24	48.0
No	26	52.0
**Total**	50	100

AJCC: American Joint Committee on Cancer; TILs: tumor-infiltrating lymphocytes

**Table 2:** summary of sociodemographic characteristics of patients with colorectal cancer

Variables	Frequency	Percentage (%)
**Age**		
20-34	10	11.1
35-49	19	21.1
50-64	40	44.4
65+	21	23.3
**Sex (n=89)**		
Male	47	52.8
Female	42	47.2
**Location**		
Colic	46	53.3
Rectum	20	22.2
Colorectal	16	17.8
Caecum	6	6.7
**AJCC stage**		
I	17	18.9
II	5	5.6
III	38	42.2
IV	30	33.3
**Treatment**		
Yes	72	80.0
No	18	20.0
**Lymphocyte infiltration**		
Yes	77	85.6
No	13	14.4
**Grade of TILs**		
Absent	13	14.4
Weak	23	25.6
Moderate	20	22.2
High	34	37.8
**Metastasis**		
Yes	52	57.8
No	38	42.2
**Total**	90	100

AJCC: American Joint Committee on Cancer; TILs: tumor-infiltrating lymphocytes

**Association between the grade of lymphocyte infiltration and chemotherapy:** Table 3 below shows that two grades of TILs in colorectal cancer patients are significantly associated with receiving chemotherapy. These grades are moderate (OR= 0.25), and high (OR= 0.13) TILs, which were inversely proportional to receiving chemotherapy before sampling. The use of chemotherapy decreases the probability of having moderate, and high TILs by 0.25, and 0.13 times, respectively. However, Table 3 also shows that there is no statistically significant association between different grades of TILs in gastric cancer patients (TIL absent, TIL high, TIL low, TIL intermediate) and chemotherapy.

**Table 3:** association between grade of lymphocyte infiltration of patients with colorectal and gastric cancers and chemotherapy

Colorectal cancer					
**Grade of TILs**	**Chemotherapy**		**OR**	[95% CI]	**p-value**
	**Yes**	**No**			
Absent	02	11	1		
Weak	00	23	0.18	0.04-0.82	0.0266
Moderate	04	16	0.25	0.08-0.74	0.0131
High	04	30	0.13	0.04-0.37	0.0002
**Gastric cancer**					
**Grade of TILs**	**Chemotherapy**		**OR**	[95% CI]	**p-value**
	**Yes**	**No**			
Absent	00	05	1		
Weak	02	06	3.00	0.60-14.86	0.178
Moderate	04	12	3.00	0.96-9.30	0.057
High	12	0.9	0.75	0.31-1.77	0.514

TILs: tumor-infiltrating lymphocytes; CI: confidence interval; OR: odds ratio

**Figure 1:**
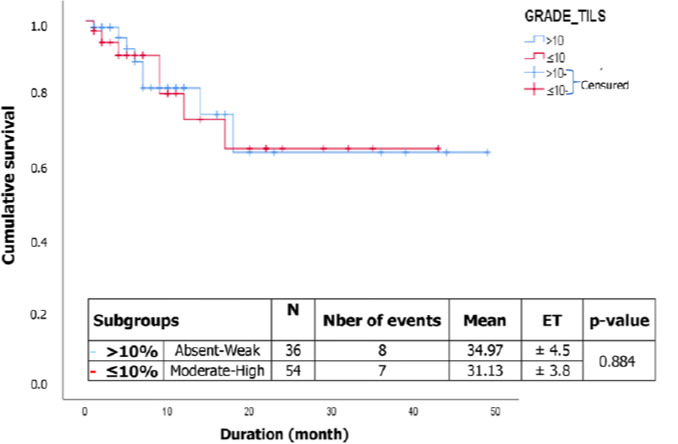
correlation between lymphocyte infiltration and survival of colorectal cancer patients

**Figure 2:**
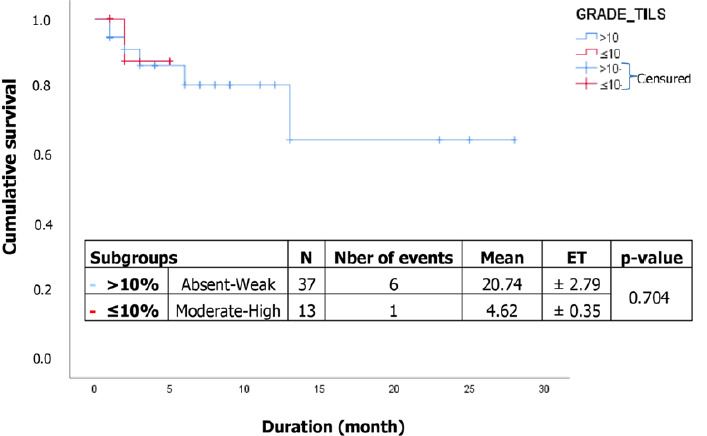
correlation between lymphocyte infiltration and survival of patients with gastric cancer

**Correlation between lymphocyte infiltration and survival:** Figure 1 shows that there was no significant difference in survival between subgroups (p= 0.884) of colorectal cancer patients. The survival estimates for the two subgroups i.e. ≤10% for absent and low TILs, and >10% for moderate and high TILs. The median survival was not reached for the different subgroups. The survival estimate is 63.3% at 49 months and 64.4% at 43 months for the ≤10% and >10% subgroups, respectively. Furthermore, Figure 2 illustrates that there was no difference in survival between subgroups (p= 0.704) of gastric cancer patients (Table 4). The survival estimate is 80.5% at 6 months for high and intermediate TILs, and 87.5% at 6 months for absent and low TILs. Patients who had metastases had a risk of death of 14.07 (aHR: 14.07, 95% CI 1.66-119.24; p= 0.015) compared with those who did not (Table 5). Similarly, patients who had not taken chemotherapy had a 21.32 greater risk of death (aHR: 21.32, 95% CI 5.35-84.96; p<0.001) than those who had (Table 5).

**Table 4:** risk factors for survival in patients with gastric cancer

Gastric cancer						
	HR	[95% CI]	p-value	aHR	[95% CI]	p-value
**Lymphocyte infiltration**						
No	1	/	/	/	/	/
Yes	0.40	0.04-3.61	0.414	/	/	/
**Grade of TILs**						
Absent	1	/	/	/	/	/
Weak	/	/	/	/	/	/
Moderate	0.87	0.09-8.68	0.904	/	/	/
High	0.28	0.02-3.19	0.307	/	/	/
**Sex**						
Male	1	/	/	/	/	/
Female	0.97	0.46-2.06	0.934	/	/	/
**Age**						
20-34	1	/	/	/	/	/
35-49	0.90	0.06-14.57	0.944	/	/	/
50-64	0.95	0.11-8.58	0.966	/	/	/
65-74	0.69	0.04-11.18	0.795	/	/	/
**Metastasis**						
No	1	/	/	/	/	/
Yes	1.08	0.50-2.32	0.840	/	/	/
**Surgery**						
No	1	/	/	/	/	/
Yes	1.00	0.12-8.48	0.996	/	/	/

HR: hazard ratio; aHR: adjusted hazard ratio; CI: confidence interval; TILs: tumor-infiltrating lymphocytes

**Table 5:** risk factors for survival in patients with colorectal cancer

	HR	[95% CI]	p-value	aHR	[95% CI]	p-value
**Lymphocyte infiltration**						
No	1	/	/	/	/	/
Yes	2.16	0.28-16.52	0.460	/	/	/
**Grade of TILs**						
Absent	1	/	/	/	/	/
Weak	2.42	0.28-20.81	0.422	/	/	/
Moderate	2.55	0.28-22.84	0.698	/	/	/
High	1.68	0.19-15.06	0.643	/	/	/
**Sex**						
Male	1	/	/	1	/	/
Female	1.59	0.53-4.75	0.406	/	/	/
**Age**						
20-34	1	/	/	1	/	/
35-49	1.60	0.17-15.45	0.684	/	/	/
50-64	1.31	0.16-11.00	0.800	/	/	/
65-74	1.74	0.18-15.78	0.621	/	/	/
**Metastasis**						
No	1	/	/	1	/	/
Yes	10.24	1.34-78.41	0.025	14.07	1.66-119.24	0.015
**Chemotherapy**						
Yes	1	/	/	1	/	/
No	14.53	4.67-45.02	<0.001	21.32	5.35-84.96	<0.001

HR: hazard ratio; aHR: adjusted hazard ratio; CI: confidence interval; TILs: tumor-infiltrating lymphocytes

## Discussion

The objective of this study was to evaluate the impact of lymphocyte infiltration on the survival of patients with digestive cancer at the Yaoundé General Hospital (YGH). We conducted a retrospective cross-sectional study over 11 months from January to November 2023, with data collection extending from July to November 2023 in the Oncology, Archives, and Anatomo-cytopathology Departments of the YGH.

The results concerning the sociodemographic characteristics of patients with gastric cancer in this study revealed an average age of 53.24 ± 12 years (range: 21-74 years). Nearly 75% of the population was older than 50 years, indicating that the study population skewed towards older individuals. These findings are consistent with those of Sylla *et al*. [5], whose study on histopathological-prognostic factors for gastric cancers reported an average age of 54 years with extremes ranging from 20 to 88 years. Men were the most represented gender, accounting for 31 cases (62%), with a sex ratio of 1.63. These results align with numerous other studies, including Ntagirabiri *et al*. [6], who investigated stomach cancer in Bujumbura and reported a sex ratio of 1.4 in favor of men. The tumor was predominantly located at the antropyloric level (46%), followed by the fundal level (44%). According to the AJCC classification, stage IV was the most common stage, representing 54% of cases. Bekolo Nga *et al*. [7] also reported similar findings in their study on prognostic factors of stomach cancer in Cameroon, with 71% of patients classified at stage IV.

Regarding the sociodemographic characteristics of patients with colorectal cancer, the mean age at diagnosis was 54 ± 14.53 years, with the most predominant age group being 50 to 64 years. This age distribution is consistent with the common occurrence of colorectal cancer in this age group. These findings are in line with the work of Arfa *et al*. [8], who reported an average age at diagnosis of 54 years in their study on the survival and prognostic factors of colorectal adenocarcinomas conducted in Tunisia. Of the patients included in our study, 47 (52.81%) were male and 42 (47.19%) were female, resulting in a sex ratio of 1.11. This study revealed a slight male predominance in colorectal cancer cases within our study population, consistent with the findings of Meddah *et al*. [9], who reported a male predominance with a sex ratio of 1.2. Colon was the primary location of tumors (53.33%), consistent with the findings of Mahamat *et al*. [10], who reported that most tumors were colonic (55.5%) in their study conducted in the Anatomo-cytopathology departments of the University Hospital Center of Yaoundé and the Pasteur Center of Cameroon. Pathological examination revealed that stage III disease extension was predominant, accounting for 42.22% of cases, suggesting that the majority of patients were diagnosed at an advanced stage. This finding is consistent with reports indicating that stage III, as defined by the AJCC, represented 41.6% of cancer cases in their study population [10].

In our study, logistic regression analysis was performed to establish an association between the grade of tumor-infiltrating lymphocytes (TILs) and chemotherapy in colorectal cancer. Two grades of TILs were significantly associated with chemotherapy use: moderate (OR = 0.25) and high (OR = 0.13) TILs. This suggests that chemotherapy inversely increases the probability of having moderate and high TILs in colorectal cancer. These findings are different from those of Fridman *et al*. [11], who demonstrated that conventional or targeted chemotherapies can induce immunogenic death of tumor cells, stimulating local immune reactions and resulting in long-term cancer control. According to this study, chemotherapy stimulates the anti-tumor immune response, thereby increasing TILs by inducing immunogenic cell death [12]. In contrast, no association was found between TILs grade and chemotherapy in gastric cancer. These results differ from those of Liu *et al*. [13], whose study focused on tumor-infiltrating lymphocytes and survival after adjuvant chemotherapy in patients with gastric cancer. In this study, patients who underwent adjuvant chemotherapy had a low TILs rate and longer disease-free survival.

In our study, the 5-year overall survival was determined for 90 patients with colorectal cancer, with 15 events (deaths) observed. Overall survival was 64.8% at 49 months in this study population, although the median was not reached. No statistically significant association was found between survival and different subgroups: ≤10% and >10% (p= 0.884). However, the survival estimate was 63.3% at 49 months and 64.4% at 43 months for the different subgroups, respectively. These findings differ from those of Jung *et al*. [14], who reported a median survival of 54 months and a statistically significant association between overall survival and high density of TILs for patients with stage III colorectal cancer in Korea.

The overall survival of patients with gastric cancer was 86.6% at 3 months. In the subgroups, survival was 86.3% at 3 months for high and intermediate TILs, and 87.5% at 3 months for absent and low TILs. No statistically significant difference was found in survival between high/intermediate and low/absent subgroups (p=0.704). The results obtained in this study are consistent with those of Zhang *et al*. [15], whose study focused on the scoring system of TILs and its prognostic value for gastric cancer, particularly for high/intermediate TILs. However, the results diverged for weak/absent TILs, with their study reporting an average survival of 78.17 ± 2.00 months for a high TILs rate compared to 41.21 ± 2.09 months for a low TIL rate.

## Conclusion

This work shows that over the last five years, the average age of patients diagnosed with colorectal and gastric cancers was 54 ± 14.53 years and 53.24 ± 11.41 years, respectively. Males were predominant in both pathologies, with a male-to-female sex ratio of approximately 1.11. Colonic location was prevalent (53%) for colorectal cancer, while the antropyloric location was predominant (46%) for gastric cancer, with stage IV being the most frequent according to the American Joint Committee on Cancer (AJCC) classification. Additionally, 86% of patients exhibited TILs in their histological samples, with a predominance of high TILs in both colorectal (38%) and gastric (42%) cancers. The efficacy of chemotherapy was inversely proportional to TILs in colorectal cancer, while no significant difference was observed between TILs and chemotherapy in gastric cancer. Furthermore, no association was found between TILs and patient survival in either colorectal or gastric cancers.

### What is known about this topic

- It has well known for many years that tumor-infiltrating lymphocytes (TILs) are associated with a favorable prognosis in patients with colorectal cancer;
- It is also known that lymphocyte infiltration is correlated with a favorable prognosis in patients with gastric cancer;
- In Cameroon, few studies have investigated the impact of lymphocyte infiltration on the survival of patients with digestive cancer.

### What this study adds

- This study demonstrates no impact of lymphocyte infiltration on the survival of patients with colorectal and gastric cancers;
- Chemotherapy performance is inversely proportional to the grade of moderate and high TILs in colorectal cancer, while no association exists between TILs and chemotherapy in gastric cancer;
- The 5-year survival rate in Cameroon between 2019-2023 was 64.8% for both colorectal and gastric cancers, indicating ongoing efforts to combat cancer in Cameroon.
